# Cardiorespiratory Fitness Cut-Points are Related to Body Adiposity Parameters in Latin American Adolescents

**DOI:** 10.3390/medicina55090508

**Published:** 2019-08-21

**Authors:** Daniel Humberto Prieto-Benavides, Antonio García-Hermoso, Mikel Izquierdo, Alicia María Alonso-Martínez, César Agostinis-Sobrinho, Jorge Enrique Correa-Bautista, Robinson Ramírez-Vélez

**Affiliations:** 1 Department of Health Sciences, Navarrabiomed-Biomedical Research Centre, IDISNA-Navarra’s Health Research Institute, Public University of Navarra, C/irunlarrea 3, Complejo Hospitalario de Navarra, 31008 Pamplona, Navarra, Spain; 2 Laboratorio de Ciencias de la Actividad Física, el Deporte y la Salud, Universidad de Santiago de Chile, USACH, Santiago 9160030, Chile; 3 CIBER of Frailty and Healthy Aging (CIBERFES), Instituto de Salud Carlos III, 28001 Madrid, Spain; 4 Research Centre in Physical Activity, Health and Leisure (CIAFEL), Faculty of Sport, University of Porto, 4200-450 Porto, Portugal; 5 Faculty of Health and Sciences, Klaipeda University, 92294 Klaipeda, Lithuania

**Keywords:** epidemiology, obesity, weight status, children, adolescents

## Abstract

*Background and Objectives:* A deficiency exists in the criterion-referenced cut-points for field-based cardiorespiratory fitness (CRF) in Latin American youths. The aims of the present study were two-fold: (1) To identify the ability of CRF estimated by the 20-m shuttle-run test (20mSRT) to differentiate between “healthy” and “unhealthy” phenotypes (by adiposity) in adolescents; (2) to assess the association between obesity and relative peak oxygen uptake (VO_2_peak) in a large and diverse sample of Latin American youths. In total, 72,505 adolescents aged between 13 and 15 years were recruited from Chile and Colombia (47.5% girls). *Materials and Methods*: The waist circumference (WC) and waist-to-height ratio (WHtR) were used to identify body adiposity markers. CRF was measured using the 20mSRT (VO_2_peak). Receiver operating characteristic curves and logistic regression were used to determine the discriminatory ability of CRF to predict body adiposity parameters. *Results:* For boys and girls, VO_2_peak showed a significant predictive capacity to detect body fat (area under the curve [AUC] > 0.62). The sensitivity of VO_2_peak was medium (>63%) for all age- and sex-specific cut-points, with optimal cut-points in 13- to 15-year olds for obesity identified as 43.77 mL·kg^−1^·min^−1^ and 38.53 mL·kg^−1^·min^−1^ in boys and girls, respectively. *Conclusions*: According to these cut-points, adolescents with low CRF were more likely to be obese either by WC or WHtR. The CRF cut-points can be used as quantitative markers for a healthier body in Latin American adolescents.

## 1. Introduction

Physical inactivity and low levels of CRF are major threats to public health. However, their prevalence is rapidly increasing in developing countries, such as among Latin American children and adolescents [1,2]. Moreover, overweight and obese children are likely to stay obese into adulthood and are more likely to develop several metabolic risks at a younger age [3]. Although the clinical manifestations of many chronic diseases mainly occur in adulthood, it is well-known that a long asymptomatic phase of development begins early in life, often during childhood [4], suggesting that chronic disease prevention initiatives should begin during these years.

CRF is an equally powerful predictor of mortality risk, similar to traditional risk factors, such as smoking, obesity, hypertension, dyslipidemia, and type 2 diabetes mellitus, in both adolescent and adult populations [5]. An important body of evidence supports an unequivocal association between poor CRF and excess adiposity with an increased risk for cardio-metabolic disease in youths [6]. In response, the fitness and health outcomes in youth assessment guidelines have called for a better understanding of the close association between component fitness and body composition [7]. In this line, the inter-relationship of higher adiposity with lower CRF is generally strongest when adiposity is measured through imaging techniques, slightly weaker when assessed using the WC and WHtR and weakest when using body mass index (BMI) [8].

Although physical fitness and body adiposity may be correlated, they are not synonymous and indicate differing disease risks. In youth studies, several authors have shown that those with low levels of adiposity and low CRF are at a greater cardiovascular risk than high-adiposity adolescents with adequate CRF, independent of muscular fitness and body composition [9,10]. Recently, Silva et al. [9] found that measurements using the 20-m shuttle run test in Canadian children aged 8–12 years were accurate to classify the sample by indicators of obesity in relation to the level of CRF. In this study, when using sex-specific cut-points, it was found that regardless of the 20mSRT indicator (VO_2_peak, laps, or speed), children with values below the recommendations were more likely to be obese, either by BMI, WC, or both, regardless of factors such as age, screen time, and level of physical activity levels. This result corroborates other studies that reported CRF to be an independent risk factor for obesity and reinforces the need for the development of CRF health-related standards to discriminate cardio-metabolic health in youths. Thus, comparing the differences in the criterion-referenced standards for the different predictive equations used to estimate aerobic capacity will help to identify different phenotype risks (optimal and poor health) from the CRF levels estimated by 20mSRT performance and will be of significant public health value. Moreover, while there are criterion-referenced cut-points for CRF in many high-income countries, only one study has been performed in Latin American countries [11].

Youth fitness assessment guidelines have called for a better understanding of the inter-relationship between physical fitness and body composition [7]. Therefore, establishing criterion-referenced cut-points associated with body adiposity parameters for adolescents can be useful to identify the target population for primary chronic diseases prevention as well as for health promotion policies. The aims of the present study were two-fold: (1) To identify the ability of CRF estimated by the 20mSRT to discriminate between “healthy” and “unhealthy” phenotypes (by adiposity measures such as WC and WHtR) in adolescents; (2) to assess the association between obesity and the relative peak oxygen uptake (VO_2_peak) in a large and diverse sample of Latin American youths.

## 2. Materials and Methods

### 2.1. Study Sample and Design

This study was based on a secondary data analysis of two separate and independent samples drawn from two different countries: Chile (n = 47,715; “System for the Assessment of Educational Quality” (SIMCE; in Spanish this translates into “Sistema de Medición de la Calidad de la Educación”, years 2011, 2012, 2013, 2015) and Colombia (n = 24,790; Prueba SER Survey, in Spanish this translates into “Evaluación en las áreas de arte, bienestar físico, convivencia y ciudadanía en el Distrito de Bogotá, Colombia” years 2014, 2015). Participants were considered eligible for this study if they were aged 13.0–15.0 years (grades 8–9), and maximal effort exercise was not contraindicated. The complete methodology of this study is described in detail [12].

Briefly, SIMCE, which has been administered annually by the Chilean Ministry of Education in November since 2011 [13], uses a proportional sample stratified across 15 geographical regions (with the exception of Eastern Island, the Juan Fernández archipelago, and the Antarctic) and three school types (public, private subsidized, and private non-subsidized). Within each stratum, schools were the primary sampling unit, and all students in selected schools were sampled. The recruitment process was carried out by the National Institute of Sport, and all examiners were technicians from this institution or graduates of physical education. Additionally, all examiners received a testing manual that described all the test procedures and protocols and completed a training course to improve the reliability and validity of the test. SIMCE certified the validity of the field test and collected the data [13]. The National Physical Education Survey was authorized under the Chilean Sports Law number 19.712, Article 5 [13]. This study was approved by the Committee on Ethics in Studies in Humans of the Institute of Nutrition and Food Technology (INTA), University of Chile, according to the norms for Human Experimentation, Code of Ethics of the World Medical Association (Declaration of Helsinki, 1995). We requested and obtained permission from the Ministry of Education to use publicly available data for research and teaching/learning purposes (available from: http://informacionestadistica.agenciaeducacion.cl/#/bases). The study authors (A.G.-H.) entered a written data use agreement with the Chilean Ministry of Education.

The Prueba SER survey was performed by Bogotá’s District Secretary of Education in November 2014 and 2015. These were cross-sectional surveys of ninth grade students recruited from public and private schools from all 20 “localidades” (municipalities) within the District Capital of Bogota (Cundinamarca Department, Andean Region of Colombia). Further details can be obtained from the website (available from: https://www.educacionbogota.edu.co/archivos/Temas%20estrategicos/Documentos/Resultados_PruebasSER-Bienestar_Fisico_Ciudadania_y_Convivencia.pdf). The survey was approved by the Review Committee for Bogotá’s District Secretary of Education (ID Convenio N° CDP 3381, Project N° 893 “Pensar en Educación” date 02-10-2014, and Fuprecol Project Code N° CEI-ABN026–000262, date 27-09-2013).

### 2.2. Body Adiposity Parameters

The measurements of weight, height, and WC were carried out at school applying standardized procedures; all the instruments were verified before measuring each subject. Before data collection, examiners were trained prior to testing by the Chilean Ministry of Education and Universidad del Rosario-Centro de Estudios en Medición de la Actividad Fisica “CEMA” (https://www.urosario.edu.co/CEMA/Inicio/#cema), to standardize the assessment process and to minimize inter-observer variability. Body mass was measured using digital weigh scales to the nearest 0.1 kg; height was measured using a stadiometer to the nearest 0.1 cm. Body mass index (BMI, kg/m^2^) was subsequently derived and BMI z-scores calculated using age- and sex-specific reference data from the World Health Organization, with obesity defined as ≥2SD above the mean [14]. Body adiposity parameters were estimated using WC and waist-to-height ratio (WHtR) information. WC was measured to the nearest 0.1 cm using an inelastic tape, which was measure positioned horizontally and midway between the lower costal border of the 10th rib and the top of the iliac crest. WC and height were used to calculate WHtR, with obesity defined as ≥0.50 according to previous reports [15]. The 75th percentile of WC according to age and gender were determined based on data collected from the de Ferranti et al. [16]. WC and WHtR have been used as proxies for central (visceral) adipose tissue which has recently received attention as a marker of ‘early health risk’ in many populations [17]. Thus, both adiposity parameters were related with cardiometabolic risks in other Latin American populations from Brazil [18] and Mexico [19], including among overweight and obese youth.

### 2.3. Cardiorespiratory Fitness (CRF)

Testing procedures were consistent with guidelines for school-based fitness assessment [7]. CRF was assessed using the 20-m shuttle-run test (20mSRT) protocol [20]. This test showed good to very good repeatability and validity [21]. The corresponding metabolic equivalent (METs) were obtained by dividing VO_2_peak by 3.5 mL·kg^−1^·min^−1^ [22]. Testing took place in the school gymnasium or on another available hard surface.

### 2.4. Statistical Analysis

Descriptive statistics were calculated for all variables (arithmetic mean, standard deviation, or frequencies). All variables were checked for normality of distribution before analysis using Kolmogorov–Smirnov test and Q-Q plots. None required transformation due to normally distributed. Cohen *d*, *t*-test, and Chi-square were computed to quantify the difference across sex. Pearson correlations were calculated to quantify the relationship between CRF and body adiposity parameters. Diagnosis screening tests [23] (sensitivity, specificity, positive predictive value (PPV), negative predictive value (NPV), positive likelihood ratio (LR+), and negative likelihood ratio (LR-)) and ROC (receiver operating characteristic) curve analysis have been applied in the determination of cut-off values for analyzed parameters [24]. However, area under curve values of 0.55–0.62, 0.63–0.71, and >0.71 corresponded to an effect size (Cohen’s *d*) small, medium, and large, respectively [25]. In addition, the present sample was classified according to the cut-points suggested in the present study and logistic regression analysis with odds ratio (OR) and 95% confidence intervals (CI) was calculated. Univariate and multivariate analysis adjusted for age and site were used separately for boys and girls. Statistical programs MedCalc 16.8.4^®^ (Ostend, Belgium) and SPSS 24.0^®^ software (SPSS Inc., Chicago, IL, USA) were used for all analyses, and a *p* value < 0.05 was considered to be statistically significant.

## 3. Results

### 3.1. General Characteristics

The final sample comprised 72,505 adolescents aged 14.02 (95% CI 14.01−14.03) years. Means (95% CIs) for the sample were: BMI, 22.65 (21.63−21.68) kg/m^2^; WC, 71.12 (71.06−71.18) cm; WHtR, 0.44 (0.44−0.44); and CRF, 42.47 (42.42−42.52) mL·kg^−1^·min^−1^ using the Léger et al. equation [20]. The prevalence of body adiposity parameters according to WC and WHtR were 11.2% and 15.1%, respectively (Table 1).

### 3.2. Cardiorespiratory Fitness Cut-Points Related to Body Adiposity Parameters

**Figure 1:**
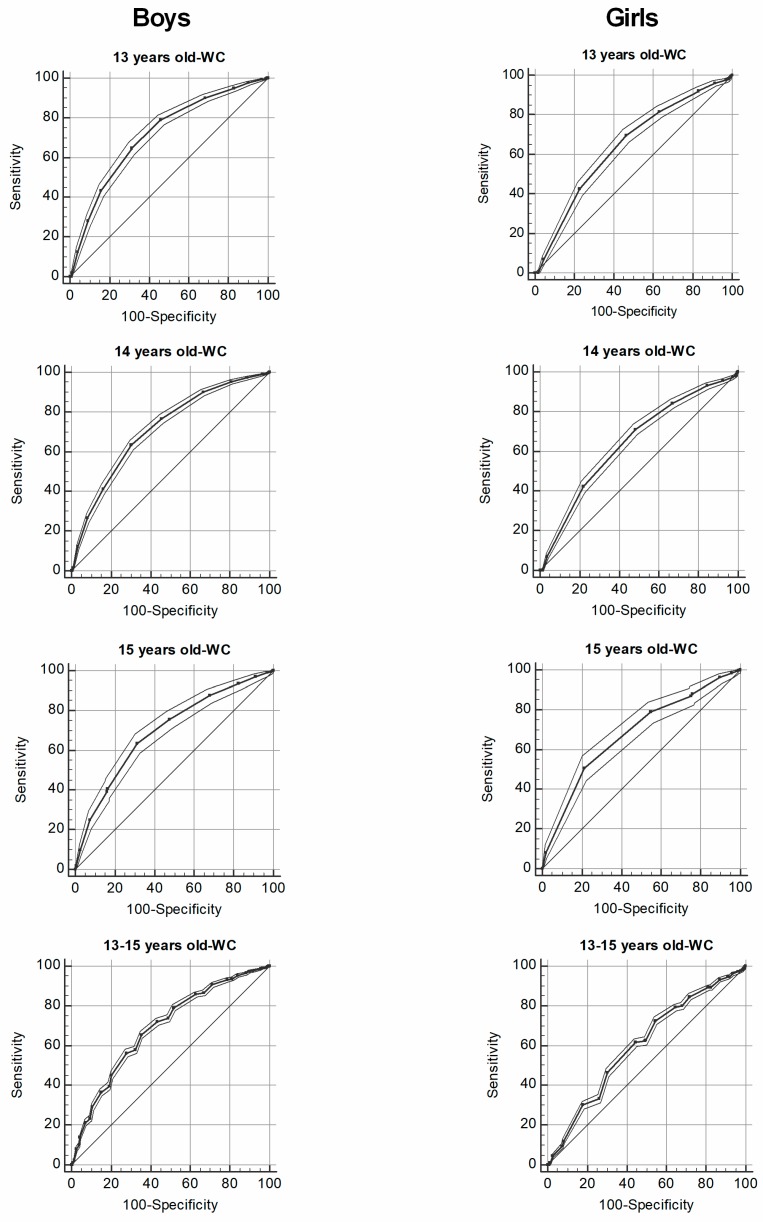
Receiver operating characteristic curve of VO_2_peak estimated by the Léger et al. equation to detect obesity by waist circumference (>75th percentile for age and sex) in boys and girls according to age.

**Figure 2:**
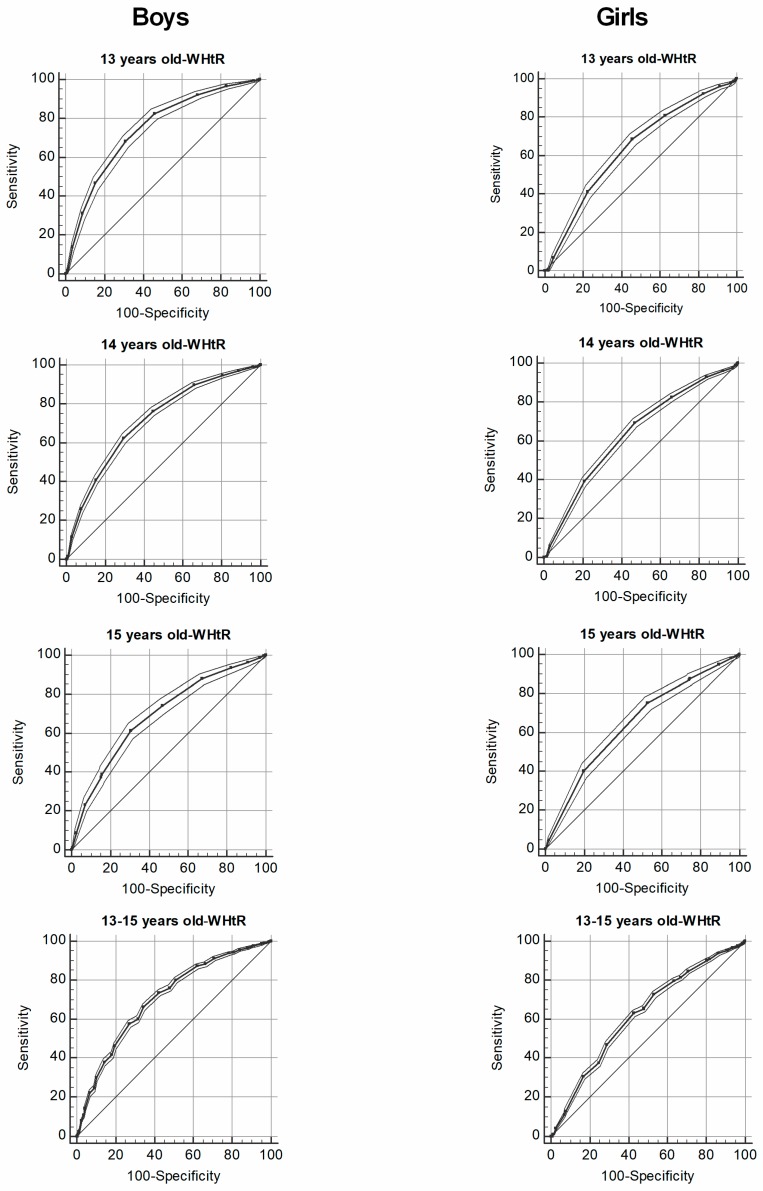
Receiver operating characteristic curve of VO_2_peak estimated by the Léger et al. equation to detect obesity by waist-to-height ratio (≥0.50) in boys and girls according to age.

For boys and girls (Table 2, Figure 1 and Figure 2, VO_2_peak showed significant predictive capacity for obesity (area under the curve, AUCs > 0.62). In boys, when considering the full sample (13–15 years), the best cut-point for VO_2_peak estimated by the equation of Léger et al. to detect body fat by WC and WHtR were 43.77 mL·kg^−1^·min^−1^ (12.51 METs). For girls, when considering the full sample (13–15 years), the best cut-point to detect body fat by WC and WHtR were 38.53 to mL·kg^−1^·min^−1^ (10.50 to 10.78 metabolic equivalent).

### 3.3. Relationship between Cardiorespiratory Fitness and Body Fat Parameters

Across all age and sex groups, CRF was weak to moderate negative correlated with WC and WHtR (Table 3).

Finally, according to cut-points suggested (Table 2), adolescents with low CRF were more likely to be obese (using BMI z-score) either by WC or WHtR (Table 4).

## 4. Discussion

The main findings of the present study suggest that CRF (estimated by the 20mSRT) presented discriminatory ability in identifying a poor health profile (estimated by body fat parameters) in both girls and boys aged 13–15 years. Additionally, the suggested cut-points for low CRF were associated with obesity in all ages and sexes.

Our study extended the results of a recent study in 8740 Canadian children aged 8.0–12.9 years that showed a negative association between the 20mSRT and cardiometabolic risk (as assessed by adiposity measures) and that the CRF cut-points from ROC analyses had a good discriminatory power for obesity [9]. In this study, the optimal suggested CRF cut-points for 8- to 12-year-olds were 39 mL·kg^−1^·min^−1^ and 41 mL·kg^−1^·min^−1^ for girls and boys, respectively. In another similar study including 16,619 British children’s from Liverpool aged 11–13.9 years [26], the values proposed to identify children at risk for overweight were 41.9 mL·kg^−1^·min^−1^ and 46.6 mL·kg^−1^·min^−1^ for girls and boys, respectively. In the present study, the optimal cut-points, when we considered all samples by WC or WHtR, were 43.77 mL·kg^−1^·min^−1^ and 38.53 mL·kg^−1^·min^−1^ for boys and girls, respectively. Another study published by our team including Colombian children and adolescents proposed cut-points of 47.9 mL·kg^−1^·min^−1^ in boys and 34.4 mL·kg^−1^·min^−1^ in girls age 9–12 years and 48.0 mL·kg^−1^·min^−1^ in boys and 33.8 mL·kg^−1^·min^−1^ in girls aged 13–17 years [11]. Thus, our results indicate that the sensitivity to identify participants with adiposity within a clinical range was limited, particularly for boys, while the specificity of the prediction of adiposity using the leger equation to estimate VO_2_peak was low for girls. In this sense, it was observed that the positive predictive value was low for both sexes aged 13–15 years (WC > 75th percentile: ♂ = 20.0 and ♀ = 13.5; WHtR ≥ 0.50: ♂ = 23.2 and ♀ = 22.8), suggesting an important number of false positives, especially in the girls group, and reinforcing the need for sex-specific targeted interventions in the Latin-American setting. This positive predictive value is directly related to the low prevalence of obesity in the overall sample (range: 6.9% to 10.8%). However, a major limitation is that cut-points can only be applied to populations in which the condition has a similar prevalence to the population tested or to individuals with a similar risk of a positive result. Furthermore, low CRF in adolescence is largely a reversible condition. Fitness-enhancing interventions in youths have been shown, on average, to improve CRF by approximately 10% [27], including among overweight and obese girls [28].

Two reviews [29,30] regarding CRF cut-points to identify red flags for healthy and unhealthy phenotypes in children and adolescents proposed interim international criterion-referenced standards of 42 mL·kg^−1^·min^−1^ and 35 mL·kg^−1^·min^−1^ for boys and girls aged 8–18 years, respectively. The cut-points proposed in our study are higher than those suggested by Lang et al. [29] and Ruiz et al. [30]; these differences may be because our study only used body fat parameters to determine a poor health profile, whereas the Ruiz and Lang cut-points used other cardiometabolic risk factors. Additionally, most of these studies used a local sample that may not have a necessary representativeness of the population. Moreover, while both studies showed cut-points for CRF for many high-income countries, we are not aware of any study using a large sample of Latin American youths.

Collectively, these findings suggest the existence of an important relationship between CRF and fat that is detectable as early as adolescence and that could be used to guide prevention efforts and, consequently, reduce cardiometabolic risk factors. The deleterious consequences ascribed to obesity could be counteracted, to some extent, by maintaining appropriate levels of CRF [6,31]. In our study, we also tested the association between CRF and fat parameters as well as the proposed cut-points of low CRF and obesity. The association between our cut-points for low CRF and obesity provide clear insight into the links between unfavourable CRF and a lower health status and highlight the need for public health interventions to increase the fitness levels in the youth population.

Such interventions—for example, in the school environment—should involve promoting physical activity to increase CRF levels [32] and were alluded to in the most recent guidelines for physical activity [33]. In this line of thought, and in agreement with our results, a systematic review [31] combining the results from 142 studies that investigated the associations between the 20mSRT and various health indicators among children and adolescents showed that the 20mSRT was positively associated with the aspects of physical, physiological, psychosocial, and cognitive health in youths and could be used as a holistic health indicator to help identify youths at risk for poor health.

Our study has several limitations that should be considered in the interpretation of the findings. First, the cross-sectional design of our study does not allow us to explain causality. Second, the assessment of other cardiometabolic risk factors (e.g., blood pressure, dyslipidemia, and insulin resistance) could have shown additional information on prognostic assessment. Finally, we predicted VO_2_peak from a single 20mSRT, and standard testing methods with directly measured oxygen consumption are required to confirm our cut-points.

The major strength of this study is the potential usefulness of the cut-points in field settings by health professionals in Latin American countries. Because the 20mSRT is a simple, low-cost method of CRF assessment, its use may provide a feasible way to study the link between physical activity and health among adolescents at the population level [34]. A final strength of this study is its large sample size, allowing the analysis to meaningfully explore various body fat parameters at the population level to better characterize Latin American adolescents.

## 5. Conclusions

Despite the cross-sectional design of the present study and that the AUC showed only moderated values, our results highlight the ability and usefulness of CRF (as estimated by the 20mSRT) in detecting fat in adolescents. Our findings also suggest that the proposed cut-points for low CRF are associated with obesity, in both sexes. Therefore, these VO_2_peak cut-points could be used by health professionals and in the school environment as a low-cost assessment in the field setting to help identify youths at risk for poor health.

## Figures and Tables

**Table 1:** Characteristics of the sample.

Characteristics	Full Sample (n = 72,505)	Boys (n = 38,044)	Girls (n = 34,461)	*p* Value (Cohen *d*) for Sex
Age (years)	14.02 (14.02−14.03)	14.05 (14.04−14.05)	14.00 (13.99−14.01)	<0.0001 (0.068)
Weight (kg)	56.01 (55.93−56.08)	57.24 (57.14−57.35)	54.64 (54.54−54.74)	<0.0001 (0.254)
Height (cm)	160.73 (160.67−160.78)	164.33 (164.25−164.40)	156.75 (156.69−156.81)	<0.0001 (1.123)
BMI (kg/m^2^)	21.65 (21.63−21.68)	21.14 (21.10−21.17)	22.22 (22.18−22.25)	<0.0001 (0.309)
BMI z-score	0.64 (0.63−0.64)	0.57 (0.56−0.58)	0.70 (0.69−0.71)	<0.0001 (0.127)
Obesity (≥+2SD) ^a^	6945 (9.6)	3852 (5.3)	3093 (4.3)	<0.0001 (N.A)
Waist circumference (cm)	71.12 (71.06−71.18)	72.11 (72.03−72.20)	70.03 (69.94−70.12)	<0.0001 (0.248)
Obesity (WC >75th percentile for age and sex) ^b^	8088 (11.2)	4489 (6.2)	3599 (5.0)	<0.0001 (N.A)
Waist-to-height ratio	0.44 (0.44−0.44)	0.44 (0.44−0.44)	0.45 (0.45−0.45)	<0.0001 (0.150)
Obesity (waist-to-height ratio ≥ 0.50) ^c^	10,897 (15.1)	5164 (7.1)	5733 (7.9)	<0.0001 (N.A)
BMI and waist circumference ^a^	4971 (6.9)	2877 (4.0)	2094 (2.9)	<0.0001 (N.A)
BMI and waist-to-height ratio ^a^	5528 (7.7)	3024 (4.2)	2504 (3.5)	<0.0001 (N.A)
Stage (n)	15.48 (15.36−15.61)	19.07 (18.86−19.28)	11.50 (11.37−11.62)	<0.0001 (N.A)
VO_2_peak (mL·kg^−1^·min^−1^)—Léger et al. [22]	42.47 (42.42−42.52)	45.83 (45.77−45.89)	38.76 (38.70−38.81)	<0.0001 (1.284)

Data in mean and 95% confidence interval or frequency and percentage. ^a^ Obesity defined as ≥+2SD according to Onis, Onyango, Borghi, Siyam, Nishida, and Siekmann [14]; ^b^ >75th percentile waist circumference definition established by De Ferranti et al. [16]; ^c^ obesity defined as ≥0.50 according to Schwandt and Haas [15]. BMI: body mass index; WHtR, waist-to-height ratio. No applicable (N.A).

**Table 2:** Diagnostic properties of VO_2_peak (20-m shuttle run test) according to the equation of Léger et al. to detect body fat by age and sex group.

Age/Sex	AUC (95% CI)	Cut-Points, mL·kg^−1^·min^−1^ and (METs)	Sensitivity (%) (95% CI)	Specificity (%) (95% CI)	LR (+) (95% CI)	LR (−) (95% CI)	PPV (%) (95% CI)	NPV (%) (95% CI)
**Obesity by WC >75th percentile for age and sex**								
13 years old								
Boys (n = 8069)	0.71 (0.70–0.72)	44.83 (12.81)	64.72 (62.3–67.0)	68.85 (67.7–70.0)	2.08 (2.0–2.2)	0.51 (0.5–0.5)	34.5 (32.8–36.2)	88.5 (87.6–89.4)
Girls (n = 8008)	0.64 (0.63–0.65)	38.53 (11.01)	69.68 (67.1–72.1)	53.63 (52.4–54.8)	1.50 (1.4–1.6)	0.57 (0.5–0.6)	23.0 (21.7–24.4)	89.9 (88.9–90.8)
14 years old								
Boys (n = 20069)	0.71 (0.70–0.72)	42.13 (12.04)	63.54 (61.5–65.5)	69.58 (68.9–70.3)	2.09 (2.0–2.2)	0.52 (0.5–0.6)	20.8 (19.8–21.8)	93.8 (93.4–94.2)
Girls (n = 18358)	0.64 (0.63–0.65)	36.74 (10.50)	71.27 (69.2–73.3)	51.78 (51.0–52.6)	1.48 (1.4–1.5)	0.55 (0.5–0.6)	14.3 (13.6–15.0)	94.1 (93.6–94.6)
15 years old								
Boys (n = 9830)	0.69 (0.68–0.70)	40.50 (11.57)	63.59 (59.7–67.4)	68.68 (67.7–69.6)	2.03 (1.9–2.2)	0.53 (0.5–0.6)	12.0 (10.9–13.2)	96.6 (96.1–97.0)
Girls (n = 7986)	0.68 (0.66–0.69)	34.95 (9.99)	79.08 (74.8–82.9)	45.22 (44.1–46.3)	1.44 (1.4–1.5)	0.46 (0.4–0.6)	7.2 (6.5–8.0)	97.6 (97.0–98.0)
13−15 years old								
Boys (n = 37968)	0.69 (0.68–0.69)	43.77 (12.51)	65.63 (64.2–67.0)	64.70 (64.2–65.2)	1.86 (1.8–1.9)	0.53 (0.5–0.6)	20.0 (19.3–20.6)	93.4 93.0–93.7)
Girls (n = 34367)	0.60 (0.59–0.60)	38.53 (11.01)	72.60 (71.1–74.1)	45.37 (44.8–45.9)	1.33 (1.3–1.4)	0.60 (0.6–0.6)	13.5 (13.0–13.9)	93.4 93.0–93.8)
**Obesity by WHtR (≥0.50)**								
13 years old								
Boys (n = 8057)	0.74 (0.73–0.75)	44.83 (12.81)	68.32 (65.9–70.7)	69.09 (68.0–70.2)	2.21 (2.1–2.3)	0.46 (0.4–0.5)	33.8 (32.1–35.5)	90.4 (89.6–91.2)
Girls (n = 8003)	0.63 (0.62–0.64)	38.53 (11.01)	68.57 (66.2–70.9)	54.13 (52.9–55.4)	1.49 (1.4–1.6)	0.58 (0.5–0.6)	26.3 (25.0–27.7)	87.8 (86.8–88.8)
14 years old								
Boys (n = 20056)	0.71 (0.70–0.72)	42.13 (12.04)	62.43 (60.6–64.2)	70.36 (69.7–71.0)	2.11 (2.0–2.2)	0.53 (0.5–0.6)	25.1 (24.0–26.1)	92.2 (91.7–92.6)
Girls (n = 18347)	0.63 (0.63–0.64)	36.74 (10.50)	69.34 (67.7–71.0)	53.22 (52.4–54.0)	1.48 (1.4–1.5)	0.58 (0.5–0.6)	22.9 (22.1–23.8)	89.7 (89.0–90.3)
15 years old								
Boys (n = 9829)	0.69 (0.68–0.70)	40.50 (11.57)	61.33 (58.1–64.5)	69.48 (68.5–70.4)	2.01 (1.9–2.1)	0.56 (0.5–0.6)	16.9 (15.7–18.3)	94.7 (94.1–95.2)
Girls (n = 7997)	0.64 (0.63–0.65)	34.95 (9.99)	75.24 (72.6–77.7)	47.12 (45.9–48.3)	1.42 (1.4–1.5)	0.53 (0.5–0.6)	18.9 (17.8–20.1)	92.1 (91.1–92.9)
13−15 years old								
Boys (n = 37942)	0.70 (0.70–0.71)	43.77 (12.51)	66.19 (64.9–67.5)	65.40 (64.9–65.9)	1.91 (1.9–2.0)	0.52 (0.5–0.5)	23.2 (22.5–23.8)	92.5 (92.1–92.8)
Girls (n = 34347)	0.62 (0.61–0.62)	38.53 (11.01)	63.99 (61.7–64.2)	57.22 (56.6–57.8)	1.47 (1.4–1.5)	0.65 (0.6–0.7)	22.8 (22.1–23.4)	88.5 (88.1–89.0)

AUC: area under the curve; 95% CI: 95% confidence interval; LR+: positive likelihood ratio; LR: negative likelihood ratio; PPV: positive predictive value; NPV: negative predictive value; WC, waist circumference; WHtR: waist-to-height ratio.

**Table 3:** Pearson correlation coefficient of association between cardiorespiratory fitness and body fat parameters by sex.

	Boys				Girls			
	13 Years Old	14 Years Old	15 Years Old	13−15 Years Old	13 Years Old	14 Years Old	15 Years Old	13−15 Years Old
WC (cm)	−0.332 *	−0.240 *	−0.179 *	−0.224 *	−0.174 *	−0.182 *	−0.193 *	−0.161 *
WHtR	−0.298 *	−0.202 *	−0.155 *	−0.203 *	−0.176 *	−0.171 *	−0.180 *	−0.154 *

WC, waist circumference; WHtR, waist-to-height ratio. * *p* < 0.001 (r, Pearson correlation coefficient).

**Table 4:** Association between unhealthy level of cardiorespiratory fitness and obesity by sex.

	Boys		Girls	
	Crude Analysis (OR (95% CI)	Adjusted Analysis (OR (95% CI) ^†^	Crude Analysis (OR (95% CI)	Adjusted Analysis (OR (95% CI) ^†^
Obesity by WC >75th percentile for age and sex				
Low cardiorespiratory fitness				
Sex-age-specific cut-points	4.18 (3.92−4.46) *	4.29 (4.01−4.59) *	2.73 (2.54−2.93) *	2.62 (2.44−2.82) *
Sex-specific cut-points	2.88 (2.70−3.07) *	4.53 (4.23−4.85) *	1.71 (1.59−1.83) *	2.73 (2.52−2.94) *
Obesity by WHtR (≥0.50)				
Low cardiorespiratory fitness				
Sex-age-specific cut-points	4.13 (3.89−4.39) *	4.58 (4.30−4.89) *	2.50 (2.36−2.66) *	2.43 (2.28−2.58) *
Sex-specific cut-points	3.24 (3.05−3.44) *	4.78 (4.48−5.10) *	2.05 (1.93−2.17) *	2.65 (2.49−2.83) *

OR: odds ratio; 95% CI: 95% confidence interval; WC, waist circumference; WHtR, waist-to-height ratio. ^†^ Adjusted analyses for age and country; * Logistic regression (*p* < 0.001).
